# The influence of climatic factors on the development of hemorrhagic fever with renal syndrome and leptospirosis during the peak season in Korea: an ecologic study

**DOI:** 10.1186/s12879-017-2506-6

**Published:** 2017-06-07

**Authors:** Yadav Prasad Joshi, Eun-Hye Kim, Hae-Kwan Cheong

**Affiliations:** Department of Social and Preventive Medicine, Sungkyunkwan University School of Medicine, 2066 Seobu-ro, Jangan-gu, Suwon, Gyeonggi-do 16419 Republic of Korea

**Keywords:** HFRS, Leptospirosis, Climatic factors, Generalized linear poisson model, Seasonality, Vector-borne disease, Rodent, Zoonosis

## Abstract

**Background:**

Hemorrhagic fever with renal syndrome (HFRS) and leptospirosis are seasonal rodent-borne infections in the Republic of Korea (Korea). The occurrences of HFRS and leptospirosis are influenced by climatic variability. However, few studies have examined the effects of local climatic variables on the development of these infections. The purpose of this study was to estimate the effect of climatic factors on the occurrence of HFRS and leptospirosis in Korea.

**Methods:**

Daily records on human cases of HFRS and leptospirosis between January 2001 to December 2009 were analyzed. The associations of climatic factors with these cases in high incidence provinces were estimated using the time-series method and multivariate generalized linear Poisson models with a maximal lag of 12 weeks.

**Results:**

From 2001 to 2009, a total of 2912 HFRS and 889 leptospirosis cases were reported, with overall incidences of 0.67 and 0.21 cases per 100,000, respectively, in the study areas. The increase in minimum temperature (1 °C) at a lag of 11 weeks was associated with 17.8% [95% confidence interval (CI): 15.1, 20.6%] and 22.7% (95% CI: 16.5, 29.3%) increases in HFRS and leptospirosis cases, respectively. A 1-h increase in the daily sunshine was related to a 27.5% (95% CI: 18.2, 37.6%) increase in HFRS at a lag of 0 week. A 1% increase in daily minimum relative humidity and a 1 mm increase in daily rainfall were associated with 4.0% (95% CI:1.8, 6.1) and 2.0% (95% CI: 1.2, 2.8%) increases in weekly leptospirosis cases at 11 and 6 weeks later, respectively. A 1 mJ/m^2^ increase in daily solar radiation was associated with a 13.7% (95% CI: 4.9, 23.2%) increase in leptospirosis cases, maximized at a 2-week lag.

**Conclusions:**

During the peak season in Korea, climatic factors play a significant role in the development of HFRS and leptospirosis. The findings of this study may be applicable to the forecasting and prediction of disease outbreaks.

**Electronic supplementary material:**

The online version of this article (doi:10.1186/s12879-017-2506-6) contains supplementary material, which is available to authorized users.

## Background

Hemorrhagic fever with renal syndrome (HFRS) and leptospirosis are common zoonotic diseases and major public health problems in rural populations in the Republic of Korea (Korea) [1, 2]. HFRS is a rodent-borne disease caused by Hantaviruses such as Hantaan virus (HTNV), Seoul virus, Puumala virus, and Dobrava virus. Rodent hosts frequently transmit viruses among themselves, as the viruses are shed in urine, saliva, and feces. Hantavirus is most often transmitted to humans via the inhalation of infectious aerosols or dried rodent excreta [1, 3]. Leptospirosis is caused by *Leptospira interrogans*, in which both rodents and domestic mammals serve as major reservoir hosts. Human infection by *L. interrogans* results from direct or indirect exposure to the urine of a host [3, 4].

The mechanisms by which HFRS and leptospirosis are transmitted are influenced by environmental, occupational, and reservoir factors [5, 6]. The relationship between meteorological variables and HFRS and leptospirosis has been assessed in many regions [7–11]. Most studies have focused on the effects of temperature, rainfall, and humidity on the incidences of HFRS and leptospirosis [8–10, 12, 13]. The prevalence of HFRS and leptospirosis in different areas depends on the viability of the pathogen in relation to the climate, human activity, landscape and seasonality [7, 8, 12, 14, 15]. The longer *L. interrogans* remains viable in the external environment, the greater the chance of its contact with susceptible hosts. The optimal conditions for the ex vivo survival of this pathogen include high humidity and a near-neutral pH [6]. In addition, outside of the host, the longevity of HTNV decreases with increasing UV radiation because the destruction of the viral lipid membrane is accompanied by a total loss of infectivity [16]. However, rodent activity increases during hot, rainy, and autumnal periods [17]. Low temperature affects not only rodent survival but also changes the behavior of mice and humans [13].

In 1951, HFRS was first recognized in soldiers during the Korean War, when more than 3200 soldiers were infected and approximately 400 died. Since its identification, HFRS has been reported globally in various clinical syndromes [18, 19]. In Korea, the HTNV and Seoul viruses are the major etiologic agents of HFRS [3, 20, 21]. The average incidence was 0.81 per 100,000 among the 3953 patients. There were 40 fatal cases between 2001 and 2010 [22]. The first reported case of leptospirosis occurred in 1984 after heavy rainfall during the harvesting season. It has also become a major public health problem [23]. Major outbreaks of leptospirosis occurred in 1984, 1985, and 1987. The estimated annual incidences from 1987 to 1991 were 0.05 to 0.11 per 100,000 during outbreak years [24], which decreased dramatically between 1991 and 1997, with an average annual incidence of 0.02 per 100,000. Between 1998 and 2011, there were 1528 reported cases, with an average annual incidence of 0.22 per 100,000 and a case fatality rate among suspected individuals of 5.8% between 2001 and 2007 [25]. Previous studies have reported pulmonary involvement to be a common outcome of leptospirosis in Korea [25, 26].

Together, these two febrile diseases and scrub typhus primarily occur during autumn in Korea [19, 27]. Leptospirosis and HFRS share common characteristics. They tend to occur during the same epidemic season and share clinical features such as capillary leakage, thrombocytopenia, hemorrhages, and altered renal function. In Korea, rodents, especially striped field mice (*Apodemus agrarius manchuricus*) and brown rats (*Rattus norvegicus*), are the main reservoirs for HFRS and leptospirosis [27, 28]. The incubation periods are also similar; i.e., 1 to 3 weeks [2, 29]. Although their incidences are decreasing, HFRS and leptospirosis are representative vector-borne infectious diseases transmitted by rodent hosts in Korea and are sensitive to climatic factors [30, 31].

Characterizing HFRS and leptospirosis regarding climatic factors will provide insight to differentiate these two zoonotic diseases on an ecologic basis. However, because of their mode of transmission and the biology of the etiologic agents, their epidemiologic features may differ. Therefore, it is essential to study the impact of climatic variables on HFRS and leptospirosis transmission because of the possibility of ensuing changes in rodent density related to climate change [8, 12, 22]. In this study, we applied empirical modeling to examine the effects of diverse climatic factors on the development of HFRS and leptospirosis during the peak season in Korea.

## Methods

### Study areas

Geographically, Korea is in a transitional zone between the continental landmass of northeast Asia and the island arc rimming the western Pacific Ocean. Its total area is 100,210 km^2^, 70% of which is occupied by mountains. The southern and western parts of the peninsula have more plains.

In general, Korea has a temperate monsoon climate with a cold winter, hot and humid summer, and sunny and dry spring and autumn seasons. January is the coldest month nationwide, with a mean temperature ranging from −5 to 5 °C. The relative humidity is the highest in July at 80 to 90% nationwide, lowest in January and April at 30 to 50%, and moderate in September and October at around 70%. The rainfall during summer is characterized by heavy showers. The rainy season lasts from late June until late July. There is also a short period of rainfall in early September. There is a crisp weather pattern in autumn (lasting from September to November), the transitional season between the hot and humid summer and the cold and dry winter months. Autumn is also the season of harvest, with the highest rodent activity.

### Data collection

National surveillance data on the daily reported cases of HFRS and leptospirosis, including information about the date of onset and location, between 2001 and 2009 were provided by Centers for Disease Control and Prevention, Republic of Korea (KCDC). As they are nationally notifiable communicable diseases (group III), the KCDC monitors these diseases. New cases of notifiable disease are reported real time to a local public health authority and information is sent to the provincial health authority and to the KCDC through an electronic reporting system [32]. Each case report contains the following information: date of onset, diagnosis, and notification, as well as patient’s age, gender, area of residence, and activities related to exposure such as harvesting and outdoor activities. Individual case information is released at the province level to protect the individual from identification under the Privacy Protection Act. This study was approved by the Institutional Review Board of Sungkyunkwan University (2015–04-017).

Meteorological data were obtained from the Korea Meteorological Agency (KMA). In 2011, the KMA had 76 weather posts across the nation, which made daily reports. These reports include the daily minimum, maximum, and average temperatures (°C); minimum relative humidity (%); daily cumulative rainfall (mm); solar radiation (mJ/m^2^); and total hours of sunshine [33]. For each case, the daily averaged weather data were matched over each province. The climatic factors were averaged across all sites in a province. The weekly averages of climatic variables were calculated based on the daily report. The annual population data of each province were retrieved from Statistics Korea [34] and divided by total provincial area [35] to estimate population density.

### Statistical analysis

**Fig. 1:**
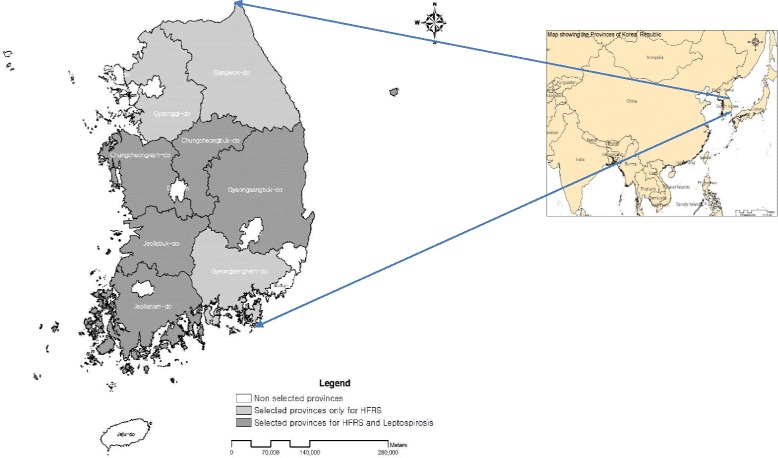
Map of geographical areas of Korea showing the eight and five provinces selected for HFRS and leptospirosis, respectively

Descriptive analyses of HFRS and leptospirosis were performed. The yearly incidences per 100,000 were calculated. Based on the highest incidences, our study primarily concentrated on non-metropolitan provinces. We selected eight provinces (Chungbuk, Chungnam, Gyeongbuk, Gyeonggi, Gyeongnam, Jeonbuk, Jeonnam, and Gangwon) for HFRS and five provinces (Chungbuk, Chungnam, Gyeongbuk, Jeonbuk, and Jeonnam) for leptospirosis (Fig. 1). Each province was characterized by four distinct annual seasons. For the purpose of analysis, weekly and monthly numbers of cases were summed in each province between January 1, 2001, and December 31, 2009, and the time series distributions of the average of climatic variables during the same period were plotted.

There is a well-established lag effect of meteorological variables prior to the initiation of HFRS and leptospirosis transmission [7, 8, 10, 11, 31]. In our study, the impacts of climatic factors on HFRS and leptospirosis among selected provinces were assessed during the peak incidence period in a year using a maximum 12-week lag between the 27th and 53rd weeks of the year. We assigned a 16-week lag duration between the 38th and 53rd weeks and 11-week lag between the 27th and 42nd weeks.

The most statistically significant variable was selected from the mean, maximum, and minimum temperatures. A multiple regression analysis with stepwise selection was performed. The R^2^ analogies and deviance were considered as the criteria for the goodness of fit. The multicollinearity of all the explanatory variables in each model was tested by variance inflation factor (VIF). We followed the recommendations of several statisticians and publications for VIF limit [36, 37], with a large VIF value of 10 or greater indicating strong collinearity.

Poisson generalized linear regression models with allowances for over-dispersion were used to quantify the effect of climatic variables on HFRS and leptospirosis [5, 8, 14, 38]. Each model was adjusted for the annual provincial population density as the dynamics of the likelihood and infection of vector-borne disease varied with host density during the study period [39]. A range of candidate set models was assessed for each lag combination and a final model was selected using Akaike information criterion (AIC). The model specifications used to assess the associations between climatic variables and numbers of HFRS and leptospirosis cases in all selected provinces for a wide range of lags in our study are as follows.

Log [E(Y)] = β_0_ + β_1_ (minimum temperature) + β_2_ (minimum humidity) + β_3_ (rainfall) + β_4_ (sunshine) + β_5_ (solar radiation) + COV

Where E(Y) is the expected number of HFRS and leptospirosis cases and β_0_ is the overall coefficient. COV represents to the population density as a confounding factor. Generalized linear model (GLM) analysis was performed using R software, version 3.0.2.

## Results

There were 2912 confirmed HFRS cases in Korea between 2001 and 2009, with an annual average incidence of 0.67 per 100,000. Similarly, there were 889 leptospirosis cases during this period, with an annual average incidence of 0.21 per 100,000. There were no statistically significant differences in HFRS and leptospirosis in patients’ gender, age, province, and occupation. Table 1 gives a general description of the HFRS and leptospirosis cases in Korea between 2001 and 2009.

**Table 1:** General description of HFRS and leptospirosis cases in Korea between 2001 and 2009

		HFRS			Leptospirosis		
		Number	Incidence^a^/(%)	*P*-value^§^	Number	Incidence^a^/(%)	*P*-value^§^
Total		2912	0.67		889	0.21	
Year	2001	293	0.62		119	0.25	
	2002	301	0.63		108	0.23	
	2003	362	0.76		107	0.22	
	2004	336	0.70		121	0.25	
	2005	328	0.68		61	0.13	
	2006	355	0.73		93	0.19	
	2007	362	0.75		155	0.32	
	2008	311	0.64		83	0.17	
	2009	264	0.54		42	0.09	
Gender	Male	1687	0.77	0.096	539	0.16	0.096
	Female	1225	0.57		350	0.25	
Age (years)	0–9	10	0.02	0.230	1	0.00	0.230
	10–19	45	0.08		5	0.01	
	20–29	173	0.25		29	0.04	
	30–39	305	0.40		53	0.07	
	40–49	475	0.65		92	0.13	
	50–59	568	1.20		160	0.34	
	60–69	733	2.24		300	0.92	
	70–79	495	2.71		212	1.16	
	80 & over	108	1.75		37	0.60	
Provinces	Seoul	199	0.22	0.242	43	0.05	0.2657
	Incheon	120	0.51		12	0.05	
	Daejeon	38	0.29		5	0.04	
	Daegu	26	0.12		9	0.04	
	Gwangju	113	0.87		44	0.34	
	Ulsan	29	0.30		9	0.09	
	Busan	54	0.17		20	0.06	
	Kangwon	143	1.07^b^		27	0.20	
	Gyeonggi	483	0.51^b^		85	0.09	
	Chungbuk	171	1.27^b^		36	0.27^b^	
	Chungnam	455	2.61^b^		100	0.58^b^	
	Gyeongbuk	269	1.12^b^		83	0.35^b^	
	Gyeongnam	152	0.54^b^		43	0.15	
	Jeonbuk	392	2.37^b^		150	0.91^b^	
	Jeonnam	263	1.56^b^		221	1.31^b^	
	Jeju	5	0.10		2	0.04	
Occupation^c^	Agricultural and fishery workers	1118	38.39%	0.229	517	58.16%	0.243
	No information	628	21.57%		128	14.40%	
	Unemployed	373	12.81%		114	12.82%	
	Housewives	255	8.76%		51	5.74%	
	Clerks	124	4.26%		23	2.59%	
	Service workers	78	2.68%		14	1.57%	
	Simple labor workers	76	2.61%		14	1.57%	
	Student	73	2.51%		10	1.12%	

^a^:/100,000/year, ^b^provinces selected for analysis, ^c^occupations selected for the calculation of percentage, ^§^Chi-square test

**Fig. 2:**
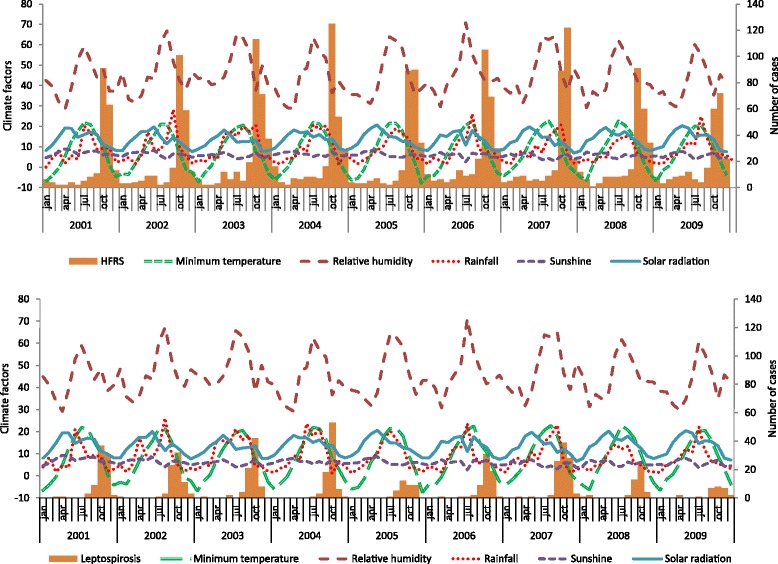
Monthly and yearly distribution of HFRS (*upper figure*) and leptospirosis (*lower figure*) cases with climatic factors in selected Korean provinces

**Fig. 3:**
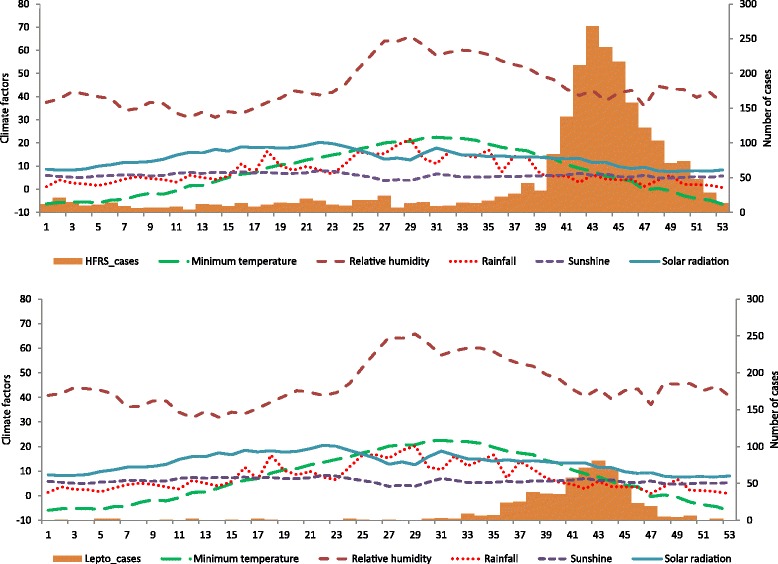
Weekly HFRS (*upper figure*) and leptospirosis (*lower figure*) cases with climatic factors from 2001 to 2009 in selected Korean provinces

Figure 2 summarizes the variation in HFRS and leptospirosis cases by month and year according to the climatic variables among the selected provinces. Although HFRS was prevalent throughout the year, there were more cases during September, October, and November than in the other months. The leptospirosis distribution pattern was similar to that of HFRS; however, there was less dispersion with the peak period. Figure 3 shows the weekly variation of HFRS and leptospirosis diagnoses according to climatic factors among the selected provinces. There was a strong seasonal variation in climatic factors and weekly diagnoses. Analysis of the weekly HFRS and leptospirosis cases among the selected provinces revealed that HFRS patients were identified every week. The numbers fluctuated weekly. The number of diagnoses began increasing after week 38, peaked at week 43, and decreased thereafter, with a slightly right-skewed distribution. In contrast, leptospirosis was not reported every week. The diagnoses increased from week 33 and peaked at week 42, with a general left-skewed distribution.

**Fig. 4:**
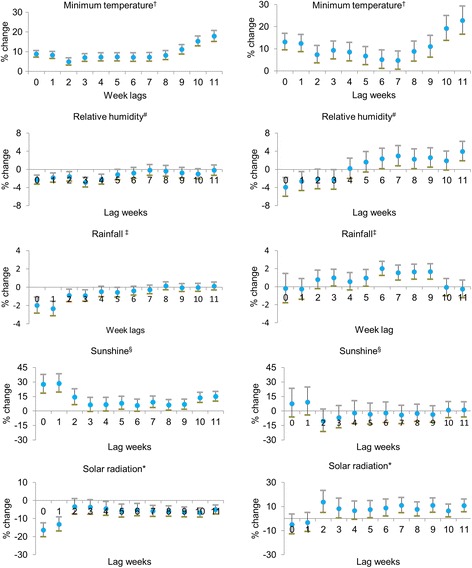
Poisson regression model of weather variables and weekly number of HFRS (*left*) and leptospirosis (*right*) cases from 2001 to 2009 in selected Korean provinces. The figure indicates the lag in weeks between HFRS and leptospirosis cases with climatic factors. The Poisson regression model shows the relation of climatic factors in 12 weekly lag durations in the development of HFRS and leptospirosis starting from week 27 to the last week of the year (53).†weekly average daily minimum temperature (°C), ^#^weekly average daily minimum humidity (%), ^‡^weekly average daily rainfall (mm), ^§^weekly average daily sunshine (hours), *weekly average solar radiation (mJ/m^2^). Percent change of risk and 95% CI were estimated using a regression coefficient (β) and the following equation: percent change of risk = (exp[β] - 1) × 100 and 95% CI = (exp[β] - 1 ± 1.96 × standard error)

The HFRS and leptospirosis diagnoses for 1 week were associated with the weather variables that occurred during the preceding weeks. Figure 4 shows estimated single-week lag effects of minimum temperature, relative humidity, rainfall, sunshine, and solar radiation on the development of HFRS and leptospirosis cases based on the final selected generalized linear Poisson regression models. The minimum temperature and sunshine had positive effects on HFRS diagnoses. However, there was negative relationship of relative humidity and solar radiation with HFRS. The weekly average minimum temperature was significantly associated with all lag times. A 1 °C increase in the weekly minimum temperature was associated with an 8.8% (95% CI: 7.1, 10.5%) increase in HFRS cases during the same week (lag 0) and a maximum increase of 17.8% (95% CI: 15.1, 20.6%) after an 11-week lag. The effect of sunshine was strongest for the development of HFRS. A 1-h increase in the average weekly sunshine was associated with a 27.5% (95% CI: 18.2, 37.6%) increase in cases during the same week and a maximum increase of 28.5% (95% CI: 19.3, 38.4) at lag 1. A 1% increase in the minimum relative humidity was associated with a 2.3% (95% CI: −3.3, −1.3%) decrease in HFRS cases during the same week and a maximum decrease of 2.9% (95% CI: −3.9, −1.8%) with a 3-week lag. The estimated lag effects of solar radiation were statistically significant for all except lags 2 and 3. A 1 mJ/m^2^ increase in solar radiation was associated with a 16.4% (95% CI: −20.2, −12.4%) maximum decrease in HFRS cases at lag 0. Rainfall was negatively associated with the development of HFRS. A 1-mm increase in the average weekly rainfall was associated with a 2.4% (95% CI: −3.2, −1.5%) maximum decrease in HFRS cases at a 1-week lag.

Regarding leptospirosis, the minimum temperature, rainfall, and solar radiation were positively associated with disease development. Like that of HFRS, there was a positive and significant overall effect of minimum temperature for all lag times. A 1 °C increase in minimum temperature was associated with a 13.1% (95% CI: 9.4, 16.9%) increase in leptospirosis cases during the same week (lag 0) and a maximum increase of 22.7% (95% CI: 16.5, 29.3%) with an 11-week lag. Rainfall also had a positive impact on leptospirosis. A 1-mm increase in the average weekly rainfall was associated with a 2.0% (95% CI: 1.2, 2.8%) increase in leptospirosis cases with a 6-week lag. A 1 mJ/m^2^ increase in solar radiation was associated with a 13.7% (95% CI: 4.9, 23.2%) increase in leptospirosis cases, maximized at a 2-week lag. There were significant associations between solar radiation and leptospirosis diagnoses from lags 5 to 11. A 1% increase in relative humidity was associated with a maximum decrease of 4.0% (95% CI: -6.0, −1.9%) at lag 0 and a significant maximum increase of 4.0% (95% CI: 1.8, 6.1%) after an 11-week lag. There was no clear effect of sunshine on leptospirosis for any lag time.

## Discussion

A key finding of this ecological study is that climatic factors are important predictors of the seasonal development of HFRS and leptospirosis in Korea. We investigated the quantitative relationship of the weekly lag impact of meteorological factors on the development of HFRS and leptospirosis. The incidences of both diseases were higher in non-metropolitan provinces than in metropolitan provinces. These diseases largely occurred during the autumn season and were more prevalent among skilled agricultural and fishery workers than in other individuals.

The incidences of HFRS and leptospirosis vary between male and female populations. This difference may reflect varying degrees of contact with the infectious rodents and their droppings between genders [5, 40]. Previous studies have reported that the incidences of HFRS and leptospirosis are male-dominated [24, 40, 41]. We also identified higher incidences of these diseases among male patients. This finding suggests that men are more likely to have contact with HFRS and leptospirosis during their daily activities and occupational exposures than women are [13, 42]. Most patients with HFRS and leptospirosis were between 50 and 79 years of age. The highest proportion of cases occurred in the 60- to 69-year age group. These findings are consistent with those of other Korean studies and suggest that older individuals are more likely to have such occupational and environmental exposures than are younger people [21, 40].

Occupational factors play an important predictive role in disease transmission. Both HFRS and leptospirosis have similar occupational trajectories [19, 40]. In our study, more than 38% and 58% of patients with HFRS and leptospirosis, respectively, were skilled agricultural or fishery workers. These individuals worked on farms where they may be exposed to Hantavirus and *L. interrogans*. Hantavirus is transmitted via aerosols that are liberated from rodent excreta (including urine and feces) [3]. In contrast, human infection with leptospirosis results from the direct or indirect exposure to the reservoir’s urine (including that of rodents, cattle, pigs, and dogs). *L. interrogans* enters the blood-stream via cuts, skin abrasions, or mucosal membranes [4, 43].

In Korea, the major rodent-borne infectious diseases (including HFRS, leptospirosis and scrub typhus) occur during the autumn season [19, 24]. There are more Hantavirus-infected rodents in the autumn than there are during other seasons of the year [44, 45]. Seasonally, there are two peaks in the antibody prevalence in striped field mice in April and between September and December. Most cases of HFRS and leptospirosis occurred during the second peak in the present study. Several factors may contribute to a higher disease incidence in autumn in Korea. This season is characterized by dry weather, increased outdoor activities such as harvesting, and increased rodent entrance into the agricultural fields [20, 27, 30, 45]. Leptospirosis in Korea is mainly transmitted by wild rodents including *Apodemus agrarius manchuricus* [23]. The risk factors for leptospirosis are influenced by occupation, host/vector climatic, and geographical and environmental characteristics of epidemiological disease patterns [23, 27, 28, 40].

We used a generalized linear Poisson model and population density to estimate the weekly lag effect of climatic factors on the development of HFRS and leptospirosis. The lag reflected delayed effect of each climatic variable on various factors associated with transmission of the infection. These factors included the proliferation of the pathogens in the external environment, seasonal fluctuations in the rodent population, human outdoor activity and pathogen infectivity to rodents and human hosts. There was a positive association between the minimum temperature and the number of cases of both HFRS and leptospirosis. The highest number was reported at an 11-week lag time, suggesting that daily low temperatures may change human and mouse behavior. Temperature is often associated with rodent survival, distribution, vegetation, and food production; therefore, it likely affects rodent densities [15, 46]. Rodents require hot and humid conditions to survive [15, 43]. Studies have shown positive correlations between temperature and rodent development, human activity, and pathogen transmission [5, 15, 43]. The highest temperatures of the year occur between June and August. There was a peak in the number of HFRS cases between September and November, suggesting that HFRS incidence lags behind the temperature by up to 3 months [34].

Relative humidity had an adverse effect on the development of HFRS during that week and up to 5 weeks earlier. Higher relative humidity has a negative impact on rodents and on the survival, infectivity, and stability of the virus ex vivo [14, 22, 47]. During the first lag weeks (0 and 1), relative humidity had a negative association with leptospirosis. This finding may result from decreased human activities during excessively humid conditions, leading to decreased *L. interrogans* exposure and transmission. The higher positive weekly lags (7, 9, and 11) suggest that *L. interrogans* requires warm, humid conditions for prolonged survival (1–2 months) outside of its host [7, 43].

Although limited rainfall is generally favorable for the transmission of vector-borne diseases [13, 15, 23], one study from eastern China reported that heavy rainfall can negatively impact rodents by destroying their habitats [13]. There was a negative impact of rainfall on the development of HFRS cases in our study. This finding may reflect decreased rodent-to-rodent and rodent-to-human contact throughout the harvesting season due to decreased rodent activity during heavy rainfall [13]. In contrast, rainfall has a positive effect on leptospirosis. In Korea, heavy rain or flooding increases the spread of infections. This is particularly true in areas with rodent inhabitation because *L. interrogans* is washed into surface waters and human behaviors may change [31]. Other studies have also reported a positive association between rainfall and leptospirosis incidence [9, 11]. In this study, there was a significant positive association between rainfall and leptospirosis at lag times of 5–9 weeks. This pattern may result from the accumulation of rainwater in water-logged soil. Rain-soaked soil is favorable for *L. interrogans* survival in the external environment [7, 9–11].

There was a significant correlation between the sunshine duration and HFRS seasonal incidence. However, the mechanisms underlying this relationship are not yet clear. The hours of sunshine were positively related to HFRS at different weekly lags. The peak duration of sunshine hours was reported in lags 1 and 2. The possible reasons for this observation include the increased flowering and seed production during mast formation due to increased sunshine that in turn facilitates an increase in the rodent population density [48, 49]. It is possible that there is an increase in HFRS transmission during long, sunny days because there is also increased outdoor human activity during this time. In contrast to HFRS, leptospirosis is not affected by sunshine. This might be the biological nature of spirochetes, as environmental conditions such as direct sunlight, dry weather and soil pH are not favorable atmosphere for the life and growth of *Leptospira* [6, 50].

Solar radiation shows opposing effects on the development of HFRS and leptospirosis. Hantaviruses are readily inactivated by heat and ultraviolet (UV) radiation, which damage viral nucleic acids and reduce their infectivity [16, 48]. Increasing solar radiation also decreases viral longevity outside of the host [16]. The survival capability of rodent-borne viruses in the external environment is critical for the transmission dynamics within the rodent populations and to humans. The longevity of these viruses outside a host might be a common adaptive characteristic; thus, they are easily transmitted even in the absence of intermediate vectors, an ability that maintains the endemic infection dynamics [51]. Unlike viruses, bacteria survive and grow well under solar radiation. Solar radiation reflects a short-term effect on bacteria. Bacteria damaged by photolytic treatment in suspension can re-grow and reactivate after the removal of solar radiation. While the solar UV radiation may inhibit the growth of bacteria, it cannot prevent the organism’s self-defense mechanism and subsequent recovery and re-growth. In a natural water source, *L. interrogans* can survive with UV-A exposure times of up to 6 h [52]. The waterborne bacterial nature of *L. interrogans* and lower doses of radiation during the rainy season may produce optimal growth conditions because the spirochetes have features unique in the microbial world, such as circular chromosomes, ribosomal gene organization, and enormous genetic diversity [53]. These characteristics might result in an increased chance of human exposure to *L. interrogans* during agricultural activities.

This study has several limitations. For instance, we did not study the influences of non-climatic factors on the development of HFRS and leptospirosis. Such factors include pathogen and host dynamics, socioeconomic status, human activities, soil and vegetation types, and vector control programs. However, these non-climatic factors are unlikely to change significantly on a weekly basis. In addition, we analyzed the KCDC surveillance data, which only include the civilian population. While military personnel are at high occupational risk for both diseases, they are not included in the KCDC surveillance data. Therefore, our findings may underestimate the true incidence of disease in Korea.

Daily HFRS and leptospirosis surveillance data were obtained from the KCDC, which manages serologically confirmed cases and weather data from the KMA. These methods allowed us to merge surveillance and weather data to observe the impact of climatic factors on the development of HFRS and leptospirosis. A GLM was used to examine the association between the weekly number of cases, the corresponding climatic factors, and the lag effect on HFRS and leptospirosis. Climatic factors influence the development of HFRS and leptospirosis differently. These results provide core information to local health authorities for the development of national infectious disease prevention and control policies.

## Conclusions

In Korea, vector-borne diseases such as HFRS and leptospirosis have multifactorial influences including patient’s occupation, meteorological variables, and human and rodent behaviors. The incidence of these diseases is seasonal, and higher in non-metropolitan provinces. Our models provide data useful for the development of an early warning system for vector-borne diseases in other regions of the world that also have seasonal disease distributions.

## Additional file

Additional file 1: Monthly sum of HFRS and leptospirosis cases with climate variables in Korea. (XLSX 658 kb)

## Abbreviations

AIC: Akaike information criterion
CI: Confidence interval
GLM: Generalized linear model
HFRS: Hemorrhage fever with renal syndrome
HTNV: Hantaan virus
KCDC: Centers for disease Control and Prevention, Republic of Korea
KMA: Korea Meteorological Agency
UV: Ultraviolet
VIF: Variance inflation factor
